# Tackling the Consumption of High Sugar Products among Children and Adolescents in the Pacific Islands: Implications for Future Research

**DOI:** 10.3390/healthcare6030081

**Published:** 2018-07-12

**Authors:** Katharine Aldwell, Corinne Caillaud, Olivier Galy, Stéphane Frayon, Margaret Allman-Farinelli

**Affiliations:** 1 Charles Perkins Centre, University of Sydney, Sydney, NSW 2006, Australia; margaret.allman-farinelli@sydney.edu.au; 2 Faculty of Health Sciences and Charles Perkins Centre, University of Sydney, Sydney, NSW 2006, Australia; corinne.caillaud@sydney.edu.au; 3 Interdisciplinary Laboratory for Research in Education, EA 7483, School of Education, University of New Caledonia, Nouméa BP R4 98851, New Caledonia; olivier.galy@univ-nc.nc (O.G.); stephanefrayon@hotmail.com (S.F.)

**Keywords:** Pacific Islands, children, adolescents, sugar, intervention, Polynesian, Melanesian

## Abstract

The Pacific Islands are experiencing an obesity epidemic with a rate of overweight and obesity as high as 80% among adults in some Pacific Island nations. Children and adolescents in the region are also affected by overweight and obesity, which is alarming due to the increased likelihood of remaining overweight as an adult. Research supports an association between poor diet and an increased risk of obesity and development of non-communicable diseases (NCDs). Excess consumption of free sugars is associated with poorer overall diet quality and increased risk of weight gain, chronic inflammation and dental caries. Traditional diets in the Pacific Islands are being supplemented with processed, high-sugar foods and beverages; thus, there is a clear need for effective interventions promoting positive dietary behaviors in the region. School and community based interventions offer an opportunity to promote positive behavior change among children and adolescents. This review aims to evaluate interventions targeting the consumption of high-sugar products in this population in the Pacific Islands.

## 1. Introduction

There are three broad geographical zones in the Pacific Islands: Micronesia, Melanesia and Polynesia [1]. The rate of overweight and obesity varies among Pacific Island countries and territories (PICTs) [2]. Tonga, Samoa and Kiribati report combined rates of adult overweight and obesity of as much as 80%, far exceeding the worldwide average [2]. Children and adolescents in the Pacific Islands are also impacted by overweight and obesity. The Global Burden of Disease study estimated a prevalence of overweight and obesity of up to 50% among boys and girls in Tonga, Samoa and Kiribati [3]. A study among nine thousand Fijian children aged 12–18 years found one quarter of participants were overweight or obese [4]. In 2011, Pacific leaders declared the obesity epidemic a health and economic crisis and threat to sustainable human development [5]. This region faces an increasing burden of non-communicable diseases (NCDs). Seven of the top ten countries/territories for prevalence of diabetes are Pacific Island nations [6]. Up to 80% of deaths in the region are related to NCDs [3,7].

Overweight and obesity and poor diet are primary risk factors for the development of NCDs [8]. Obesity during childhood and adolescence increases the likelihood of remaining above a healthy weight as an adult [8,9]. Childhood obesity is associated with several health conditions including increased risk of fracture, breathing difficulty, hypertension, early markers of cardiovascular disease (CVD), insulin resistance and depression [8]. The transition from childhood to adolescence involves increased independence, and formation of long-term dietary and lifestyle habits. Therefore, poor dietary behaviors developed during this period can significantly impact lifelong health [10,11]. Addressing poor dietary behaviors, before they become long-term habits, may prove effective in promoting sustainable behavior change and preventing weight gain [12].

Among various dietary factors related to weight gain, excess consumption of free sugars has been extensively researched. The term “free sugar” (sometimes referred to as added sugar) is the sugars added to foods and beverages during production and processing, in addition to the sugars naturally found in honey, syrups, fruit juices and fruit juice concentrates [13]. Current recommendations state that free sugars should comprise no more than 10% of total energy intake [14]. However, global dietary assessment studies suggest children and adolescents regularly exceed this recommendation [13,15,16,17]. Increased modernization, urbanization, economic development and market globalization in the Pacific Islands have driven a rapid nutrition transition towards consumption of processed and packaged foods [18,19,20,21]. Nations have become increasingly reliant upon imported produce, such as canned and preserved foodstuffs which has negatively impacted nutritional quality [20]. The rise of trans-national corporations and increased exposure to global food markets in the Pacific Islands has driven a nutrition transition towards consumption of more Westernized, processed foods and beverages [18,21,22]. The traditional Pacific diet is based on starchy root vegetables, such as taro or yams, and starchy fruits, such as breadfruit and banana. Other staples include seafood, non-starchy vegetables and other fruits [18]. This traditional dietary pattern contains little processed products and is low in free sugars. PICTs have become increasingly reliant on imported products high in free sugars. These products compete with domestic produce that have higher production costs and are less convenient [18,23]. Polished rice, processed breads and snack products are replacing traditional staples, meats are replacing seafood, and high sugar processed snacks are replacing fruit [24]. Studies among children and adolescents in PICTs have reflected this nutrition transition.

Excess consumption of free sugars has been linked with poor oral health, weight gain, chronic inflammation and the development of NCDs [25,26,27,28,29]. The significant increase in poor oral health among Pacific Islanders has been partially attributed to increased consumption of processed imported high sugar products [25]. Urban communities have shown higher prevalence of dental caries, which may be due to the ease of access to high-sugar products compared to those living in remote communities [25]. Sugar sweetened beverages (SSBs) have been a focus point for research with a large body of evidence supporting a relationship between excess consumption of free sugars and weight gain in both children and adults [14,27,28,30,31]. Decreased intake of dietary sugars is associated with significant weight loss and reduction in pro-inflammatory markers. Reducing consumption of SSBs and high-sugar products in early childhood and adolescence may have longer-term health benefits [29,31]. A small number of published dietary assessment studies in the Pacific Islands suggest consumption of SSBs and high-sugar snacks in this population is common, while fruit and vegetable intake is inadequate [17]. Interestingly, PICTs with higher rates of adolescent overweight and obesity have reported significantly higher rates of SSB consumption [19]. A study conducted in six Pacific Island nations involving more than ten thousand school-going adolescents found 40% of participants consumed one or more soft drinks per day. Only half of participants consumed the recommended serves of fruit and only one in three participants consumed more than three serves of vegetables per day [32]. Soft drink consumption was also associated with overweight and obesity [32]. Research in Tonga and Fiji has shown high-sugar foods and beverages are readily available and easy to access at school canteens [33]. Coupled with the intrinsic reward of sugary food and drinks (i.e., the taste of these products), the increased availability of high-sugar products can significantly enhance the acceptability and desirability of these products [34]. Clearly, effective strategies are needed to target consumption of products high in free sugars. 

School and community based interventions may promote positive dietary behavior changes in children and adolescents. However, across the Pacific region, there is limited information on strategies that have been implemented in this population and whether they were successful in improving dietary behavior and reducing the risk of overweight and obesity. The aim of this review was to identify and evaluate the outcomes of school or community based programs that involved strategies targeting consumption of high-sugar products among children and adolescents in the Pacific Islands.

## 2. Methods

A literature search was conducted utilizing online research databases: Medline, Global Health, Science Direct and PubMed. A combination of search terms was used to identify relevant articles. Search terms are summarized in Table 1.

School or community based interventions that involved strategies to reduce consumption of high-sugar foods and beverages among children and adolescents were included. Studies were excluded if they were conducted outside of the Pacific Islands or targeted adults. Studies were also excluded if they didn’t provide results of the intervention.

## 3. Results

Four published studies were identified that targeted consumption of high-sugar products among children and adolescents in the Pacific Islands. A summary of these studies is provided in Table 2. Overall, it was very difficult to identify studies that provided evaluations of outcome measures. Those that were identified included a small controlled trial in French Polynesia, an evaluation of Fiji’s National School Canteen Guidelines and two larger studies from the Tongan and Fijian arms of the Pacific Obesity prevention in Communities (OPIC) program. Firstly, in 2005, the Fiji Ministries of Health and Education, and National Food and Nutrition Centre released the National Food and Nutrition Policy for Schools and School Canteen Guidelines. Schools were instructed to promote healthier school environments. For example, instructions were given on monitoring the quality of students’ lunches to ensure they were ‘healthy’. School canteens also provided healthy meals containing seasonal, fresh produce and remove unhealthy items (including SSBs and snacks high in free sugars and fats) from shelves [35,36]. Mandatory compliance to these guidelines began from early 2009. An evaluation of Fijian schools in 2010 found less than one fifth of assessed schools were compliant with guidelines [37]. Students who attended schools that were classed as ‘adherent’ were significantly less likely to be overweight or obese than students who attended ‘non-adherent’ schools [36,37]. 

One of the key intervention features of the Tubuai Island College intervention was to improve the school canteen environment by offering healthy alternatives to high-sugar products [38]. Compared to control groups, those exposed to the intervention had a significantly reduced rate of weight gain [38]. The Ma’alahi Youth Project in Tonga and the Healthy Youth Healthy Communities project in Fiji were part of the OPIC program. These projects utilized multiple strategies including modifications to the school environment, education programs, activities in schools and policy changes. Results from both studies were not significant in reducing weight gain or promoting reduced consumption of high-sugar products [39,40,41].

## 4. Discussion

This review found a very small number of published school and community based programs that involved strategies targeting consumption of high-sugar products among children and adolescents in the Pacific Islands. Overall, the quality of the studies that were available was quite poor. Furthermore, while other interventions may have been implemented in this region, it is impossible to assess their impact due to the absence of quality, long-term publications of outcomes. Similar conclusions were drawn from a recent study evaluating school and community based NCD prevention programs worldwide [42].

The largest studies identified from the literature search originate from the Pacific OPIC program [39,40,41,43,44]. The OPIC program was originally conducted between 2004 and 2009. Its primary aims were to promote healthy eating (including reduced SSB and sugary snack consumption), physical activity and healthy weight in adolescents [43,44]. Four nations were involved in the project: Fiji, Tonga, New Zealand and Australia. A large component of the program involved using a community capacity building approach to promote healthy behaviors among communities in various settings including schools and churches. Community capacity building aims to enhance knowledge, skills, structures and systems in the environment to increase the ability of a community to deliver and sustain implementation of a program [40]. Unfortunately, available evaluations assessing the impact of the program suggest it was largely unsuccessful in reducing overweight and obesity and did not result in sustained healthy behavior changes among children and adolescents [39,40,41,43,44]. The OPIC program was limited by a lack of good quality, preliminary information to guide the development program components for each country [43,44]. One significant challenge identified by OPIC researchers was that the capacity of research staff was overstretched. This impacted the quality of data collected, and the records and results that were kept [44]. For example, the Healthy Youth Healthy Communities (HYHC) study in Fiji lost 59% of participants at follow-up, severely compromising data quality [40,41]. Furthermore, preliminary investigative studies looking at factors, such as socio-cultural influences on diet and lifestyle, were completed alongside the OPIC intervention components rather than prior to development of the study [43,44]. This reduced the capacity of researchers to consider the unique determinants of dietary behaviors in the study population. Exploring these determinants may have enabled researchers to target their strategies more effectively.

Various factors are known to determine dietary intake in children and adolescents. This includes individual characteristics, social and physical environmental influences and societal influences. Firstly, individual characteristics, such as attitudes, beliefs and knowledge, influence food choice [34,45]. There is limited data available on the perception of high-sugar foods and beverages in children and adolescents in the Pacific Islands. Studies in this population worldwide suggest the perceived ‘healthiness’ of a product may influence consumption [46]. This is important to note considering the way that many high-sugar products, such as sports drinks and energy drinks, are marketed. This age group often takes words and phrases commonly used in advertisements and on food packaging at face value (e.g., ‘natural’, ‘refreshing’ and ‘hydrating’) [47]. Addressing misconceptions about the healthiness of high-sugar foods and beverages may reduce desirability of these products. Self-perceived body weight and self-awareness of being overweight are also critical for weight loss and positive dietary changes [48]. A study among New Caledonian adolescents found half of overweight and obese adolescents underestimated their weight [48]. When coupled with factors, such as lack of access to healthy, traditional foods and beverages, this perception may result in increased intake of high-sugar products with poor nutritional quality [33].

Other individual characteristics, such as taste and preference, dominate food and beverage choices of children and adolescents [47]. The taste, texture and smell of high-sugar products are appealing and often increase their desirability [34]. However, this influence can be overridden by regulating access to these products [49]. Research in Tonga and Fiji has shown high-sugar foods and beverages are readily available and easy to access at school canteens [33]. While healthy canteen guidelines have been developed in some Pacific Island nations, such as Fiji, implementation and adherence to guidelines thus far has been limited [37]. It is up to governing bodies including ministries of health and national departments to ensure that school health policies are adhered to. Several other countries, such as the Cook Islands and Tonga, report having school food policies in place, but there are no published data on their impact, which makes it difficult to draw conclusions about the success or benefit of such programs [50]. A recent systematic review found that school nutrition policies targeting the reduction of SSBs significantly decreased consumption of SSBs among school students [51]. Interestingly, interventions that regulated access to SSBs in the school environment had the highest success rate in terms of reducing consumption of SSBs [51]. A review in the United States also found that students who attended schools with marketing bans on SSBs or limited availability in school canteens were less likely to drink these products [52]. However, another study from the United States found that SSB consumption was not significantly different. Interestingly, when availability was regulated at school, students compensated by drinking more SSBs outside of the school [53]. 

While regulating access to SSBs and high-sugar products in school may be an effective strategy, accessibility in other environments, such as the home, can also strongly influence intake. An Australian study in 2014 found that the strongest predictor of soft drink consumption among school aged children was availability in the home [54]. Including parents in future programs may facilitate positive changes in the home to reduce access to high-sugar products. This strategy has been successful in other countries, for example, the Health in Adolescents program in Norway. This program involved parents, providing strategies for improving accessibility and availability of healthy options at home. The program resulted in a significant reduction in SSB consumption and improvement of other dietary behaviors [55]. Providing access to healthy alternatives is vital to ensure positive dietary changes in this population. Research indicates that while many Pacific Island people prefer locally produced foods, they consume inferior imported products due to social and economic barriers to access [23]. The low cost and convenience of high-sugar products has promoted increased intake. The Tubuai Island College trial showed that providing access to healthier locally produced foods reduced the rate of weight gain in adolesecents [38]. Making healthy options, such as fresh fruits and vegetables available, must be incorporated into future programs.

Delivering interventions in school and community based settings, may be an effective means of promoting sustainability, facilitating intervention delivery and capacity for research outputs. For example, teachers or community members can be educated and engaged to facilitate components of an intervention. This can encourage maintenance of a program and balance the commitment of research members, teachers and the community. By utilizing local members of the community, researchers may also gain further insight into cultural or social trends that may influence the dietary habits of children and adolescents in the area. This method has been utilized with some success in other regions of the world, such as, the C3 program in the United States and the Dutch Obesity Intervention in Teenagers study. Both interventions utilized lessons targeting diet and lifestyle behaviors during class time with significant reductions in the consumption of SSBs for both studies [56,57]. Schools also offer the opportunity for social support. As a children transition into adolescents, peers may become more influential on dietary choices [58]. Interventions that involve collaboration with peers may be an effective strategy to promote behavior change. The fluids used effectively for living (FUEL) study in Canada used a peer educator approach to promote reduced consumption of SSBs among participants. Peer led groups significantly reduced their intake of SSBs compared to control groups at 3 months follow up [59]. Interventions that involve behavior change techniques and build self-management skills, such as self-monitoring, may also improve health behaviors including consumption of high-sugar products. One of the most frequently used behavior change techniques used in interventions is providing information about the health consequences of performing the behavior. Educational programs that explain to adolescents the consequences of excess consumption of free sugars via SSB and processed snack consumption may be beneficial. Educating children, while they are young about these consequences, may increase awareness and instill positive behaviors at earlier ages [51].

Consumption of SSBs and high-sugar products is not uniform across children and adolescents in the Pacific Islands. For example, a recent New Caledonian study found children and adolescents of Melanesian backgrounds and those living in a rural area were more likely to regularly consume SSBs [60]. Adolescents residing in rural locations were also more likely to be overweight or obese [60]. It is important that future programs target ‘at risk’ groups. Treatment programs for children and adolescents with obesity should also be considered along with obesity prevention programs. A large proportion of children and adolescents in the Pacific Islands are already above a healthy weight. Current evidence suggests that weight status can be improved through a variety of dietary regimes, including low carbohydrate, low fat or increased protein diets, intermittent fasting and even very low energy diet plans for adolescents with obesity [61]. While there is no ‘one size fits all’ dietary pattern for weight reduction, most of these diets ultimately result in reduced overall energy intake and reduction in consumption of free sugars [61].

Finally, while school and community based programs may promote healthy dietary habits, governments should also be responsible for implementing upstream strategies to improve the overall nutrition environment. Adopting taxes on the importation and sale of high-sugar products, such as SSBs, seems a beneficial strategy for decreasing the access to and availability of such products. Several nations have implemented taxes on SSBs and high-sugar foods (e.g., confectionary and processed snacks). Samoa has both import and domestic excise taxes on soft drinks applied since 1984. In Samoa, bottled water is now cheaper than soft drinks [20,50,62]. In 2002, French Polynesia introduced local and import taxes on all SSBs, confectionary and ice cream [50,62]. In 2006, Fiji placed an import tax and domestic excise tax on soft drinks. However, in 2007, domestic taxes were reduced, due to lobbying from the domestic soft drink industry [62]. In 2007, Nauru introduced a 30% import tax on sugar, confectionary, carbonated soft drinks, cordials, flavored milks and drink mixes [50,62]. Unfortunately, the impact of these taxes is largely unknown due to a lack of evaluation studies. A review of Samoan policies identified consumption of soft drinks in adults slightly decreased between 1991 and 2003 [62]. This suggests the taxes may have positively influenced dietary behavior; however, recent studies from the region are unavailable [62]. Conducting a large-scale evaluation of sugar taxes in the Pacific region would be valuable to determine whether these strategies are effective in producing positive behavior change in addition to producing revenue. Large-scale public health campaigns that involve digital communications and social media, such as the ‘Change4Life’ social marketing program in the United Kingdom, would also be beneficial. One key promotion in this campaign was ‘smart swaps’, which encouraged substitution of sugary drinks for non-sugar, diet drinks, milk or water. Purchase data from the promotional time period showed an 8.5% reduction in purchase of carbonated SSBs [13]. Implementing similar campaigns in the Pacific Islands may result in raised public awareness and reduced purchase of SSBs and high-sugar products.

The lack of quality published evaluations of school and community based programs implemented among children and adolescents in the Pacific Islands limits the ability to conclude which strategies may be useful to reduce the consumption of free sugars in this population. There is little good-quality longitudinal data from cohort studies among children and adolescents in the Pacific Islands, which makes it difficult to explore common dietary trends and compare to health outcomes in this population. While studies among children and adolescents worldwide offer insight into aspects that influence dietary behaviors, it would be useful to further evaluate differences between gender, age, ethnicity and geographic location in this population. Identifying whether there are any differences between these factors will allow for future programs to tailor strategies more effectively.

## 5. Conclusions

Children and adolescents in the Pacific Islands are at risk of developing a range of non-communicable diseases due to the rate of overweight and obesity in this region. Current research suggests this population frequently consumes free sugars via SSBs and high sugar snack foods. Research to date suggests there has been little success from school and community based interventions in reducing the consumption of high-sugar products in this population. The results of previous programs have highlighted the importance of conducting comprehensive preliminary assessments among target populations to inform the development process. To conclude, a combination of strategies ranging from school policies to nutrition education and behavior change interventions should be used to tackle the ongoing problem of excess consumption of free sugars among children and adolescents in the Pacific Islands. Governments have a responsibility to ensure health policies are adhered to and to implement other strategies, such as sugar taxes and public health initiatives, to achieve success in these programs.

## Figures and Tables

**Table 1:** Search terms used to generate relevant studies.

Topic	Term
Dietary exposure	Sugar OR added sugar OR sugar sweetened beverages, OR processed foods
Health	Overweight OR obesity
Setting	School OR community
Population	Children OR adolescent(s) OR Pacific Islands OR Polynesian OR, Melanesian OR Micronesian
Behavior	Behavior OR dietary behavior OR behavior change
Other	Intervention OR education OR nutrition education OR promotion

**Table 2:** Summary of identified school and community based programs among children and adolescents in the Pacific Islands.

Authors	Name of Study	Participants	Intervention/Strategy	Results
Varman S. et al. (2013) [37]	-	230 Fijian primary schools	Healthy canteen guidelines regulating availability of energy-dense nutrient-poor foods and providing access to healthy products. The study sites were 230 primary schools in Fiji’s Western Division.	33 (14%) schools had no canteen data. Of the 197 schools with canteen data, 31 (14%) schools were fully compliant
Gatti C. et al. (2015) [38]	Tubuai Island College Intervention	Intervention group: School students aged 10–18 years (*n* = 240). The group was divided based on attendance status: external collegians, half residents and full residents. Control group: School students aged 10-18 years (*n* = 90) from a neighboring island	5-month controlled trial set in the Tubuai Island college in French Polynesia. School nutrition program: aimed to offer healthier foods in the school canteen. Participants also received information on healthy lifestyles. Food sellers surrounding the college were encouraged to promote fruit, water and diet drink sales. Parents could also attend several sessions before, during and after the program to advice parents about benefits of healthy lifestyles. Physical activity program: 2–4 h of canoe training was implemented each week. Intervention groups were exposed to the intervention differently based on their attendance status. For example, external collegians did not eat in the school canteen whereas full residents at all meals at the school canteen. Control group received no intervention.	Weight increased significantly in all groups except for residents after 5 months of follow up. Intervention group had a significantly lower rate of weight gain than controls. Control group: adjusted weight gain was 4.2 kg (95% CI, 3.4–5.0) after 5 months. Intervention group adjusted difference in weight change was −3.4 kg (95% CI, −4.3 to −2.5). The proportion of adolescents who lost weight increased (*p <* 0.001) with exposure to healthy food and physical activity.
Fotu K.F. et al. (2011) [39]	Ma’alahi Youth Project	School students aged 11–19 years. (Intervention group at baseline *n* = 1083 and follow up *n* = 815. Comparison group at baseline *n* = 1396, follow up *n* = 897)	Tongan arm of the OPIC project. Intervention was conducted in three districts of the main island of Tongatapu (Houma, Nukunuku and Kolonga). School students attending all six secondary schools on the island of Vava’u were used as a comparison. Used social marketing approaches and community capacity building. Included school policies, community breakfasts, vegetable gardens, infrastructure provisions and activities, such as fun runs. Content varied by location. Baseline data collected between September 2005 and March 2006 with second baseline data collection in February and March 2007. Follow up data was collected between April and December 2008.	No significant difference in weight, BMI or prevalence of overweight/obesity between intervention and comparison groups. Adjusted weight (kg) difference was 0.05 (*p* = 0.89) and adjusted BMI difference was −0.02 (*p* = 0.36). Adjusted body fat percentage difference was -1.46 (*p* < 0.001). Intervention participants reported increased intake of SSBs (significantly greater than comparison groups).
Kremer P. et al. (2011) [40]	Healthy Youth Healthy Communities (HYHC)	Adolescents aged 13–18 years (Intervention group at baseline *n* = 2670 and follow up *n* = 879. Comparison group at baseline *n* = 4567 and follow up *n* = 2069)	Fijian arm of the OPIC project conducted over three school years (2006–2008) but with total of just over 2 years actual intervention exposure. Intervention conducted in Nasinu and three towns on the western side of Viti Levu were used as comparison regions. Multiple sites including faith-based organizations and schools. Intervention strategies included policy changes, education programs, and activities in schools and infrastructure changes. Content varied by location.	No significant difference in weight or BMI between intervention and comparison groups. The intervention group also reported poorer quality of life at follow up. Adjusted differences in weight (kg) and BMI were 0.05 (*p* = 0.81) and 0.10 (*p* = 0.13), respectively.
